# Age and Witnessed Apneas as Independent Predictors of Obstructive Sleep Apnea After Stroke: A Prospective Cohort Study

**DOI:** 10.3390/jcm14238332

**Published:** 2025-11-24

**Authors:** Michela Figorilli, Marta Melis, Chiara Cualbu, Giulia Frongia, Federico Arippa, Stefania Redolfi, Monica Puligheddu

**Affiliations:** 1 Sleep Disorder Research Center, Department of Medical Sciences and Public Health, University of Cagliari, 09124 Cagliari, Italy; m.figorilli@aoucagliari.it (M.F.); mrtmelis@gmail.com (M.M.);; 2 Neurology Unit, Azienda Ospedaliero-Universitaria, Monserrato, 09042 Cagliari, Italy; 3 Department of Medical Sciences and Public Health, University of Cagliari, 09124 Cagliari, Italy; 4 Department of Mechanical, Chemical and Materials Engineering, University of Cagliari, 09124 Cagliari, Italy

**Keywords:** stroke, obstructive sleep apnea, screening model, sleep monitoring, secondary prevention, sleep-disordered breathing

## Abstract

**Background:** Obstructive sleep apnea (OSA) is frequent but underrecognized after stroke, worsening prognosis, recurrence, and mortality. Polysomnography is rarely feasible in acute care, and existing screening tools have limited accuracy. We aimed to identify simple OSA clinical predictors to improve risk stratification in stroke patients. **Methods:** In this prospective study, 116 consecutive acute stroke patients (mean age 73 years, 57% male) underwent standardized clinical evaluation, Berlin Questionnaire, Epworth Sleepiness Scale (ESS), and home sleep apnea test during hospitalization. OSA was defined as apnea–hypopnea index (AHI) ≥ 15. Logistic regression identified independent predictors; the model’s performance was assessed by accuracy, sensitivity, specificity, and ROC curves. **Results:** OSA was diagnosed in 42 patients (36%). OSA patients showed higher NIHSS at admission (*p* = 0.048) and higher ESS scores (*p* = 0.047), but similar vascular risk factors and stroke subtypes compared to non-OSA patients. In a multivariate analysis, age (OR 1.05; 95% CI 1.00–1.10; *p* = 0.036) and witnessed apneas (OR 6.20; 95% CI 1.31–29.22; *p* = 0.021) were OSA independent predictors. The two-variable models achieved 72.9% accuracy, 90.3% specificity, 41.2% sensitivity, Nagelkerke R^2^ = 0.223, and AUC = 0.739 (*p* < 0.001), outperforming both the Berlin Questionnaire (AUC 0.596) and ESS (AUC 0.616). **Conclusions:** A simple model based on age and witnessed apneas reliably identified stroke patients at high risk for OSA, with good discriminative performance and higher accuracy than standard questionnaires. Its high specificity supports targeted allocation of sleep studies in resource-limited acute settings, potentially improving early detection, secondary prevention, and care pathways after stroke.

## 1. Introduction

Obstructive sleep apnea (OSA) is highly prevalent among stroke patients, with estimates ranging from 29% to 72% depending on study design and diagnostic criteria [1,2,3]. Beyond its high frequency, OSA is associated with worse prognosis, including cognitive decline, functional disability, longer hospitalization, increased recurrence, and higher mortality [4,5]. RCTs have shown that treatment of poststroke OSA using continuous positive airway pressure (CPAP) improves neurologic recovery and quality of life, as well as reducing daytime sleepiness and depressive symptoms [6,7]. Overall, OSA detection and management may be an important strategy to improve poststroke outcomes.

Despite this clinical relevance, OSA often remains underdiagnosed in stroke patients. Polysomnography (PSG), the diagnostic gold standard, is rarely performed in routine stroke care due to logistics and economic barriers [8]. Home sleep apnea testing (HSAT) is a feasible alternative, but access and interpretation still limit its widespread use. Consequently, there is a growing need for pragmatic, clinically feasible screening approaches that can be integrated into acute stroke care.

Several predictive models and screening tools—such as the Berlin Questionnaire [9], the Epworth Sleepiness Scale (ESS) [10], STOP-BANG [11], and other composite scores—have been proposed for OSA risk assessment in stroke. However, their performance has been modest, with reported areas under the curve ranging between 0.55 and 0.82. Moreover, many models suffer from methodological biases, lack of external validation, and reliance on anthropometric variables that are often unavailable or unreliable in acute neurological settings [12,13,14,15].

To address these limitations, simplified and easily applicable approaches are warranted. In this prospective cohort study, we aimed to identify clinical and sleep-related OSA predictors in consecutive stroke patients, with the goal of constructing a pragmatic model to improve risk stratification and guide the selection of patients who would most benefit from sleep monitoring in the stroke unit.

## 2. Materials and Methods

### 2.1. Study Design and Population

We conducted a prospective observational study including consecutive patients admitted for acute stroke to the Neurology Unit, Azienda Ospedaliero-Universitaria, Cittadella Universitaria di Monserrato, SS 554 KM 0.700, 09042 Monserrato (CA), Italy between January 2023 and December 2024. Eligible participants were adults (≥18 years) with a confirmed diagnosis of ischemic stroke, hemorrhagic stroke, or transient ischemic attack (TIA).

Exclusion criteria included refusal to provide informed consent and inability to complete sleep questionnaires despite caregiver assistance. The study was approved by the local Ethics Committee (“Comitato Etico Sardegna”, protocol number PNRR-MAD-2022-12375812, 1 November 2022). Written informed consent was obtained from all participants or their caregivers, in accordance with the Declaration of Helsinki.

### 2.2. Clinical Assessment

Baseline demographic and clinical variables included age, sex, body mass index (BMI), length of hospitalization, comorbidities, and vascular risk factors (smoking, hypertension, diabetes, dyslipidemia, atrial fibrillation, previous stroke or myocardial infarction, heart failure, and chronic kidney disease). Neurological evaluation comprised the Italian version of the National Institutes of Health Stroke Scale (NIHSS) and was performed at admission and at discharge. The modified Rankin Scale (mRS) was collected pre-stroke and at discharge. The NIHSS is a tool to assess stroke severity, with scores ranging from 0 (no stroke) to 42 (profound stroke). The scale comprises 11 elements evaluating functions such as consciousness, vision, and motor control, with higher scores indicating more severe neurological impairment [16]. The mRS is a widely used 7-point scale (0–6) that measures the degree of disability or dependence in daily activities after stroke or other neurological events, assessing functional independence, with high scores indicating more severe disability [17]. Stroke etiology was classified according to the TOAST criteria [18]. The presence of patent foramen ovale (PFO), atrial fibrillation (AF), extracranial or intracranial atherosclerosis, embolic stroke of undetermined source [19], stroke in young patients (<60 years), and wake-up stroke were also recorded.

### 2.3. Sleep Assessment

All patients underwent standardized sleep evaluation using the Berlin Questionnaire and the Epworth Sleepiness Scale (ESS). The Berlin Questionnaire has been extensively applied as a screening tool for OSA on a global scale. It comprises 11 items grouped into three categories addressing (1) presence, frequency, and severity of snoring and presence of witnessed apneas; (2) unrefreshing sleep, fatigue or sleepiness behind the wheel; and (3) history of arterial hypertension or obesity (BMI greater than 30 kg/m^2^). A positive score in two or more categories indicates a high risk for OSA: this metric was used in the present study [9]. The ESS has been widely used to assess subjective daytime sleepiness, evaluating the propensity to doze (0: none; 1: mild; 2: moderate; 3: severe) during 8 daily life activities. An ESS score > 10—applied in the present study—indicates excessive daytime sleepiness [10].

Home sleep apnea testing (HSAT), polygraph type III (Embla MPR PG, Natus Medical Inc., 3150 Pleasant View Road, Middleton, WI 53562 USA) was performed in all patients during hospitalization following the American Academy of Sleep Medicine (AASM) recommendations [8] in collaboration with the Sleep Disorder Research Center, University of Cagliari. The following parameters were recorded: nasal pressure and oral thermistor for oronasal flow assessment; thoracic and abdominal respiratory inductance plethysmography belts for respiratory efforts evaluation; and for detection of thoraco-abdominal asynchronism or paradox detection, pulse-oximetry, 1-lead EKG, and body position. A trained physician scored polysomnographic recordings manually using the Remlogic software (version Embla 3.4.1). Respiratory events were scored using the latest AASM criteria [20] as follows: apnea was defined as a drop of at least 90% of airflow from baseline, lasting 10 s or longer, while hypopnea was defined as a ≥30% drop of airflow lasting at least 10 s, associated with a ≥3% oxygen saturation drop. Extracted parameters included total recording time, percentage of total recording time spent in supine position, apnea–hypopnea index (AHI), oxygen desaturation index (ODI), mean SpO_2_, mean desaturation amplitude, time with SpO_2_ < 90% (T90%), and mean heart rate. OSA was defined as an AHI ≥ 15 events/hour, consistent with the AASM guidelines [21], in order to identify those stroke patients at the highest risk, where targeted interventions are most likely to yield meaningful clinical benefits.

### 2.4. Statistical Analysis

Continuous variables were expressed as mean ± standard deviation (SD), and their differences between OSA and non-OSA groups were assessed using Student’s *t*-test or Mann–Whitney U test, as appropriate. Categorical variables were expressed as frequencies, and their distribution across groups was assessed using χ^2^ or Fisher’s exact test. A *p*-value < 0.05 was considered statistically significant. To identify OSA independent predictors, we performed a logistic regression analysis with OSA as the dependent variable. Variables with *p* < 0.10 at univariate analysis, together with those deemed clinically relevant for OSA identification, were considered for entry in the multivariate logistic regression model. A total of eight candidate predictors were initially entered into the multivariable analysis (age, sex, BMI, NIHSS at admission, hospitalization length, fatigue, unrefreshing sleep, and ESS score), corresponding to an events-per-variable ratio (EPV) of 5.25. A backward stepwise (Wald) method was applied, with entry and removal criteria set at *p* = 0.05 and *p* = 0.10, respectively. Odds ratios (ORs) with 95% confidence intervals (CIs) and associated *p*-values are reported. A *p*-value < 0.05 was considered statistically significant. To ensure model transparency and adherence to the TRIPOD guidelines, model assumptions and diagnostics were evaluated [22]. Multicollinearity was assessed using Variance Inflation Factors (VIFs), with values < 2 considered acceptable. The linearity of the relationship between age and the logit of OSA probability was verified by including a quadratic term (age^2^). Age was also rescaled per 10-year increase (age/10) to improve clinical interpretability. Model calibration was assessed using the Hosmer–Lemeshow test. Model performance was evaluated through classification accuracy, sensitivity, specificity, and receiver operating characteristic (ROC) curve analysis. ROC curves were generated both for the final regression model and for clinical screening tools (Berlin and ESS), and their areas under the curve (AUC) were compared. Optimal thresholds were determined using the Youden index (J = Sensitivity + Specificity − 1). For each instrument, the optimal cut-off, sensitivity, specificity, PPV, NPV, accuracy, and AUC were calculated and compared with the regression model.

Sensitivity analyses were then performed to assess the robustness of the predictive model by applying alternative AHI thresholds (≥5 and ≥30 events·h^−1^) to define OSA. Using the same set of predictors retained in the main AHI ≥ 15 model, logistic regressions were re-estimated, and ROC curves were generated to evaluate model discrimination and classification performance. Internal validation (e.g., bootstrap resampling) and interaction testing (e.g., age × sex) were not conducted due to sample size constraints and software limitations. All statistical analyses were performed using SPSS version 26 (IBM Corp., Armonk, NY, USA).

## 3. Results

### 3.1. Population Characteristics

**Figure 1:**
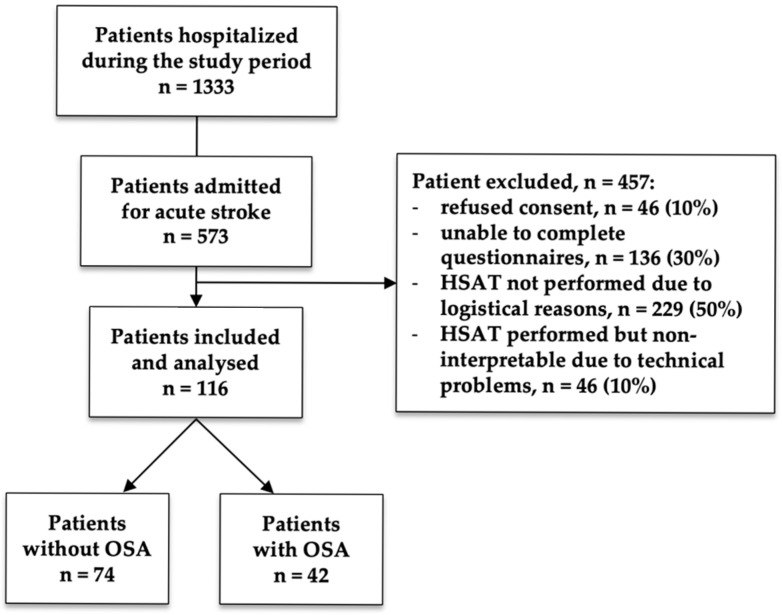
Patient flowchart.

As shown in the flow chart in Figure 1, a total of 1333 patients were hospitalized during the study period. Of these, 573 were admitted for acute stroke and were screened for eligibility. A total of 457 patients were excluded from the final analysis for the following reasons: (1) refusal to provide informed consent, including both patients who actively refused to participate (7%) and patients whose caregivers refused on their behalf (3%), (2) inability to complete the study questionnaires due to acute clinical conditions, such as severe neurological impairment (e.g., high NIHSS score 19%), cognitive impairment preventing completion (e.g., aphasia 7%), or acute medical instability (e.g., critical stroke complications 4%), (3) home sleep apnea test (HSAT) not performed due to organizational issues (e.g., lack of available equipment or staff 27%), patient unavailability for testing (e.g., discharge before scheduled test 17%), or test interrupted due to technical issues or patient discomfort (6%), and (4) HSAT performed but non-interpretable due to technical problems (e.g., sensor malfunction 6%), poor data quality (e.g., excessive movement during testing 2%), or insufficient recording time (2%). The excluded patients were older (80 vs. 73 years, *p* = 0.0003), had higher pre-stroke disability (mRS 1 vs. 0, *p* = 0.0001), and experienced longer hospital stays compared to those included (*p* = 0.03), while no significant differences were observed in stroke severity or comorbidities.

Ultimately, 116 patients were included in the final analysis. The mean age was 73 years, with a slight male predominance (57%). The majority of HSAT recordings were performed in the acute period, generally within 10–14 days from stroke onset. The overall prevalence of OSA (AHI ≥ 15) was 36% (42 patients). OSA patients tended to be older and have higher BMI than those without OSA, although these differences did not reach statistical significance. The distribution of vascular risk factors (smoking, hypertension, diabetes, dyslipidemia, atrial fibrillation, coronary artery disease, heart failure, and chronic kidney disease) was similar across groups (Table 1).

### 3.2. Stroke Characteristics

The distribution of stroke subtypes did not significantly differ between patients with and without OSA (Table 2). However, NIHSS score at admission was significantly higher in OSA patients compared to controls (3.6 ± 3.3 vs. 2.5 ± 2.2, *p* = 0.048). No significant differences were observed in NIHSS at discharge, mRS scores, or hospitalization length, although a trend toward longer hospital stay was noted among OSA patients (13 ± 15 vs. 9 ± 6 days, *p* = 0.070).

### 3.3. Sleep Assessment

As expected, OSA patients exhibited markedly worse sleep study parameters, including higher AHI (35 ± 15 vs. 6 ± 4), higher ODI (37 ± 15 vs. 7 ± 5), lower mean SpO_2_ (92% vs. 93%), and longer T90% (23% vs. 6%). Among the clinical symptoms, witnessed apneas showed a trend-level association in the univariate analysis (*p* = 0.065), later confirmed as an independent predictor in multivariable modeling. Epworth Sleepiness Scale scores were significantly higher in OSA patients (6.7 ± 3.9 vs. 5.1 ± 3.7, *p* = 0.047), whereas the proportion of patients with ESS > 10 or a positive Berlin Questionnaire did not significantly differ (Table 3).

### 3.4. Multivariate Analysis

In the multivariate logistic regression model which includes age, sex, BMI, NIHSS at admission, hospitalization length, fatigue, unrefreshing sleep, ESS score, only age (OR = 1.05; 95% CI 1.00–1.10; *p* = 0.036) and witnessed apneas (OR = 6.20; 95% CI 1.31–29.22; *p* = 0.021) remained independent OSA predictors (Table 4). Although NIHSS was associated with OSA in univariate analysis, it did not retain significance in the multivariable model once age was included, suggesting partial overlap in the clinical information captured by the two remaining variables. Multicollinearity diagnostics confirmed the absence of significant correlations among predictors (all VIF < 2). Age linearity with the logit of OSA probability, tested by including a quadratic term (age^2^), was non-significant (*p* = 0.57). Model calibration was good, as indicated by a non-significant Hosmer–Lemeshow test (χ^2^ = 11.4, df = 8, *p* = 0.18). The final model achieved an overall classification accuracy of 72.9%, with high specificity (90.3%) but modest sensitivity (41.2%), and a Nagelkerke R^2^ of 0.223, indicating a moderate explanatory power.

### 3.5. ROC Analysis

**Figure 2:**
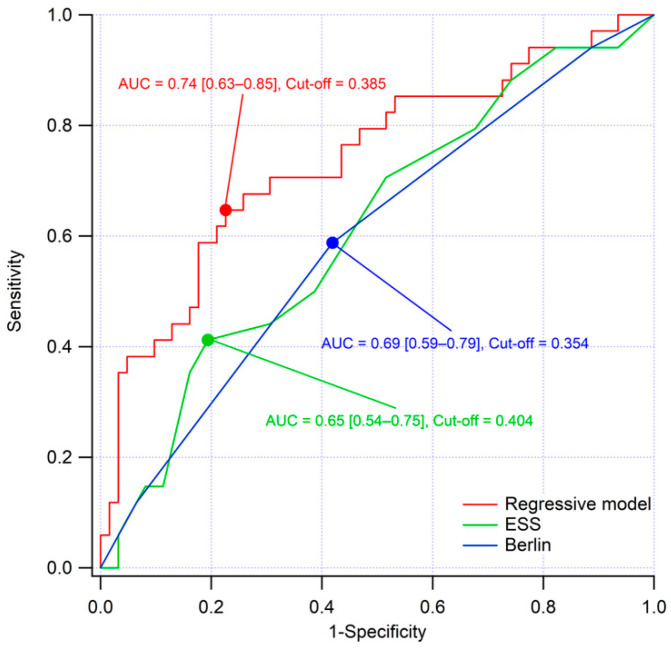
Receiver operating characteristic (ROC) curves comparing the predictive performance of the multivariate logistic regression model, the Berlin Questionnaire, and the ESS for the identification of OSA (AHI ≥ 15) in stroke patients.

ROC curve analysis confirmed the discriminative ability of the model, with an AUC of 0.739 (95% CI 0.630–0.847; *p* < 0.001). In contrast, the Berlin Questionnaire (AUC = 0.596, *p* = 0.120) and the ESS (AUC = 0.616, *p* = 0.062) showed lower and non-significant accuracy (Figure 2). Overall, the proposed regression model outperformed both questionnaires in identifying OSA among stroke patients. At the optimal Youden-derived thresholds, the cut-offs were 0.4039 for ESS, 0.3537 for Berlin, and 0.3851 for the regression model. The regression model achieved the highest Youden index (J = 0.421) compared with ESS (J = 0.218) and Berlin (J = 0.169), confirming a superior discriminative ability and a more balanced trade-off between sensitivity and specificity. The corresponding performance metrics, including sensitivity, specificity, PPV, NPV, and accuracy, are reported in Supplementary Table S1.

The model performance remained consistent across alternative diagnostic thresholds. AUC values were 0.702, 0.738, and 0.888 for AHI ≥ 5, ≥15, and ≥30, respectively. As expected, lowering the diagnostic threshold increased sensitivity (89.4%) while reducing specificity (22.6%), whereas higher thresholds yielded greater specificity (93.7%) with moderate sensitivity (38.9%). The corresponding performance metrics, including AUC, sensitivity, specificity, PPV, NPV, and accuracy, are summarized in Table S2 and Figure S1 of the Supplementary Materials.

To allow for model verification and external replication, the anonymized minimal dataset is provided as Supplementary Material (Dataset S3). It includes only those de-identified variables required for replication of the main analyses. No information allowing patient re-identification is included.

## 4. Discussion

### 4.1. Main Findings

In this prospective cohort of consecutive stroke patients, we found that age and witnessed apneas were the most robust independent OSA predictors, achieving an overall accuracy of 73%. Importantly, the predictive model demonstrated high specificity (90%) but modest sensitivity (41%). These findings highlight that simple, easily obtainable variables can provide clinically relevant risk stratification in acute stroke settings, where access to PSG or even home sleep apnea testing (HSAT) is frequently limited. Our regression model exhibited better discriminative performance compared with the Berlin and ESS questionnaires, as reflected by the higher AUC and the strongest overall classification balance. The model’s performance also remained stable across alternative AHI thresholds (AHI ≥ 5 and ≥30), further supporting the robustness of this approach. As expected, lowering the diagnostic threshold increased sensitivity at the expense of specificity, whereas higher thresholds yielded greater specificity with only moderate sensitivity.

### 4.2. Comparison with Previous Literature

Our findings are consistent with the high prevalence of OSA reported in stroke patients, estimated to affect up to 72% of this population [1]. Prior models have typically relied on combinations of demographic, clinical, and anthropometric variables such as BMI, neck circumference, and waist circumference [12,13,14,15]. However, their predictive performance has been variable, with AUCs ranging from 0.55 to 0.82, and most studies have suffered from small sample sizes, methodological biases, and lack of external validation [12,13,14,15].

The review by Yang et al. (2024) emphasized these limitations and underlined the need for pragmatic models that eliminate dependence on anthropometric measurements, often unavailable in acute stroke care [23]. In this regard, our results are novel in demonstrating that a two-variable model (age + witnessed apneas) is sufficient to outperform traditional questionnaires such as the Berlin Questionnaire and the ESS, both of which showed low discrimination in our cohort.

Furthermore, our observation that witnessed apneas are a stronger predictor than questionnaire-derived risk scores is in line with prior evidence showing that traditional screening tools often misclassify patients because symptoms such as fatigue and sleepiness overlap with stroke-related manifestations [9,10,11,13,14]. By contrast, caregiver-reported witnessed apneas may provide a more objective indicator of nocturnal breathing abnormalities. The primary strength and innovation of this study lies in the development of a two-variable model that integrates age and witnessed apneas to identify stroke patients at high risk for sleep apnea. This model is a feasible and easily implementable bedside tool that can be integrated into the routine stroke care.

### 4.3. Clinical Implications

The association between OSA and stroke is well established, as stroke patients have a markedly higher risk of developing OSA than the general population [1,3]. This increased susceptibility reflects the impact of stroke on respiratory control and upper airway stability: brainstem injury can impair respiratory drive, while stroke-related reductions in muscle tone and swallowing dysfunction further promote airway obstruction. In addition, vascular and autonomic alterations—such as cerebral ischemia and disrupted autonomic regulation—can destabilize upper airway tone, collectively increasing the likelihood of airway collapse during sleep [24].

The results presented in our study have a direct relevance for the daily practice in stroke units. The identification of age and witnessed apneas as the most reliable OSA predictors suggests that a very simple model can guide clinicians in selecting which patients should undergo respiratory monitoring. Because both variables are rapidly obtainable during clinical assessment, this model can be applied even in acute settings where time and resources are limited. Its high specificity indicates that patients flagged as high-risk are very likely to have OSA, which may help reduce unnecessary sleep studies and focus diagnostic resources on those most likely to benefit from it. Most importantly, early OSA recognition and treatment in the stroke population are crucial. In fact, untreated OSA has been consistently associated with poorer functional outcomes, higher recurrence rates, and increased mortality [4,5]. By enabling more targeted screening, this pragmatic model may therefore contribute to more effective secondary prevention and improved stroke care pathways.

The fact that our regression model retained better performance even when all classical tools were evaluated at their optimal threshold conditions reinforces the notion that post-stroke OSA may be better captured through objective clinical information rather than symptom-based questionnaires, whose diagnostic accuracy is inherently limited in the acute phase. Interpreting these findings also requires consideration of the clinical meaning of OSA severity thresholds. While the model achieved its highest accuracy when OSA was defined as AHI ≥ 30, AHI ≥ 15 is the most appropriate primary outcome as it identifies moderate-to-severe OSA, the range for which treatment is most consistently recommended in current guidelines. By contrast, AHI ≥ 30 isolates only the most severe cases and would risk excluding patients with moderate OSA who may still benefit substantially from targeted evaluation and treatment. Thus, the improved performance at AHI ≥ 30 reflects the easier discrimination of severe disease rather than a more appropriate threshold for clinical screening. In this context, the model’s consistent discriminative ability across thresholds supports its potential use as a pragmatic tool for prioritizing which stroke patients should undergo further sleep evaluation.

Although the sensitivity of our model was modest (41%), its high specificity (90%) has clear clinical relevance in the acute stroke setting. In real-world hospital environments, where time, staff, and equipment availability often limit the number of sleep studies that can be performed during hospitalization, a highly specific model allows clinicians to confidently prioritize patients at greatest risk of OSA—typically older individuals and those with witnessed apneas—for in-hospital HSAT. This targeted screening strategy ensures that limited diagnostic resources are allocated to patients most likely to have clinically significant OSA, optimizing efficiency without compromising care quality. For those not identified as high-risk, clinicians can recommend post-discharge home-based sleep testing, ensuring continuity of diagnostic evaluation once acute management is complete. In this sense, our model functions as a practical triage tool rather than a comprehensive diagnostic instrument, aligning with the operational realities of stroke units. Its implementation could facilitate an efficient two-step approach—in-hospital HSAT for high-probability cases and outpatient follow-up for the remainder—thereby improving detection rates, supporting secondary prevention, and promoting more personalized management of post-stroke patients.

### 4.4. Limitations

Several limitations should be acknowledged. First, this was a single-center study with a moderate sample size, which may limit generalizability; also, I even if we did not exclude hemorrhagic strokes specifically, we were unfortunately unable to recruit any patients with this stroke subtype.

Second, using HSAT-III rather than PSG may have led to an underestimation of hypopnea prevalence. Still, HSAT-III remains a valid and practical tool for identifying moderate-to-severe OSA in hospitalized stroke patients. Because full PSG is often unfeasible in acute care settings, we used an AHI ≥ 15 to capture clinically significant OSA and reduce the risk of missing high-risk cases. This approach provides a balanced compromise between diagnostic accuracy and resource limitations, making it suitable for routine stroke-unit practice.

Third, we did not include biomarkers, echocardiographic variables, or neuroimaging data, which have been proposed as promising predictors in previous studies [15].

Fourth, given that eight predictors were initially considered and only 42 OSA events were available, the EPV fell below the commonly recommended threshold of 10, indicating a potential risk of overfitting, which should be considered when interpreting the final model. Internal validation procedures should also be implemented in future studies to further assess model robustness and clinical utility.

Finally, while the model performed well in our cohort, because of all the limitations listed so far, further validation in larger, independent, and multicenter cohorts is necessary to confirm the generalizability of the model.

### 4.5. Future Directions

Although promising, our findings require validation before they can be widely implemented. Future studies should aim to confirm the predictive performance of this model in larger, multicenter cohorts that include more diverse patient populations and different healthcare settings. In addition, integrating other predictors—such as biomarkers of systemic inflammation, echocardiographic parameters, or neuroimaging features—may improve sensitivity and overall accuracy, particularly for patients with milder forms of OSA that remain clinically relevant. Another important avenue is the adaptation of the model to different phases of stroke, as the prevalence and detectability of OSA may vary between acute and chronic stages. Finally, the translation of this model into a digital tool, such as a mobile application or an electronic health record-embedded calculator, could facilitate bedside implementation and support standardized OSA screening in stroke units. In the longer term, the combination of such pragmatic models with artificial intelligence approaches may further optimize early detection and resource allocation, ultimately improving patient outcomes.

## 5. Conclusions

In summary, this study demonstrates that a simple, pragmatic model based on age and witnessed apneas enables the identification of ischemic stroke patients at increased risk of OSA with good accuracy, outperforming traditional questionnaires. Although sensitivity remains limited, the model’s high specificity supports its use as a targeted screening strategy to optimize resource utilization in stroke units. With further validation and digital translation, this approach has the potential to improve early OSA detection and strengthen secondary prevention after stroke.

## Figures and Tables

**Table 1:** Demographic and clinical characteristics of the overall sample and comparison between patients without OSA (AHI < 15) and those with OSA (AHI ≥ 15). Data are presented as mean ± SD and n (%). Abbreviation: AHI, apnea–hypopnea index.

	All (n = 116)	AHI < 15 (n = 74)	AHI ≥ 15 (n = 42)	*p*-Value
Age, years	73 ± 13	71 ± 14	75 ± 10	0.145
Female	50 (43)	34 (46)	16 (38)	0.441
Body Mass Index, kg/m^2^	27 ± 5	26 ± 5	28 ± 4	0.093
Smoking	29 (25)	17 (23)	12 (29)	0.512
Hypertension	82 (71)	50 (68)	32 (76)	0.398
Diabetes	27 (23)	17 (23)	10 (24)	1.000
Dyslipidemia	85 (73)	52 (70)	33 (79)	0.387
Atrial Fibrillation	28 (24)	19 (26)	9 (21)	0.658
Coronary Artery Disease	11 (9)	7 (10)	4 (10)	1.000
Heart Failure	6 (5)	4 (5)	2 (5)	1.000
Chronic Kidney Disease	4 (3)	2 (3)	2 (5)	0.620

**Table 2:** Stroke characteristics of the overall sample and comparison between patients without OSA (AHI < 15) and those with OSA (AHI ≥ 15).

	All (n = 116)	AHI < 15 (n = 74)	AHI ≥ 15 (n = 42)	*p*-Value
Recurrent Stroke	16 (14)	8 (11)	8 (19)	0.296
Wake-up Stroke	35 (30)	24 (32)	11 (26)	0.533
Transient Ischemic Attack	6 (5)	4 (5)	2 (5)	1.000
Stroke in Young Patients	18 (16)	13 (18)	5 (12)	0.594
Patent Foramen Ovale	4 (3)	4 (5)	0 (0)	0.296
Extracranial Atherosclerosis	67 (58)	40 (54)	27 (64)	0.335
Congruent * Extracranial Atherosclerosis	24 (21)	12 (16)	12 (29)	0.151
Intracranial Stenosis	7 (6)	4 (5)	3 (7)	0.701
Congruent * Intracranial Stenosis	5 (4)	3 (4)	2 (5)	1.000
Embolic Stroke of Undetermined Source	23 (20)	14 (19)	9 (21)	0.814
NIHSS (admission)	2.9 ± 2.7	2.5 ± 2.2	3.6 ± 3.3	0.048
NIHSS (discharge)	1.4 ± 2.0	1.2 ± 1.5	1.8 ± 2.7	0.124
mRS (pre-Stroke)	0.4 ± 1.1	0.5 ± 1.3	0.3 ± 0.9	0.441
mRS (admission)	1.1 ± 1.1	1.1 ± 1.5	1.1 ± 1.4	0.992
Hospitalization Length, days	10.5 ± 10.2	9 ± 6	13 ± 15	0.070

Data are presented as mean ± SD and n (%). Abbreviations: AHI, apnea–hypopnea index; *: located in an artery congruent with the territory of the index stroke; NIHSS, National Institutes of Health Stroke Scale; mRS, modified Rankin Scale. Bold indicates *p*-value < 0.05.

**Table 3:** OSA characteristics of the overall sample and comparison between patients without OSA (AHI < 15) and those with OSA (AHI ≥ 15). Data are presented as mean ± SD and n (%). Abbreviations: % supine, % of total recording time spent in supine position; AHI, apnea–hypopnea index; ODI, oxygen desaturation index; SpO_2_, pulsed oxygen saturation; T90% percentage of total recording time spent with SpO_2_ < 90%; bpm, beats per minute; ESS, Epworth Sleepiness Scale. Bold indicates *p*-value < 0.05.

	All (n = 116)	AHI < 15 (n = 74)	AHI ≥ 15 (n = 42)	*p*-Value
**Sleep Study:**				
-Total recording time, min	539 ± 102	533 ± 107	548 ± 93	-
-% supine	58 ± 31	55 ± 31	63 ± 30	-
-AHI	17 ± 17	6 ± 4	35 ± 15	-
-ODI	18 ± 18	7 ± 5	37 ± 15	-
-Mean SpO_2_, %	93 ± 3	93 ± 3	92 ± 2	-
-Mean Desaturation Amplitude, %	4.8 ± 3.1	4.1 ± 1.5	6.1 ± 4.6	-
-T90%, %	13 ± 21	6 ± 17	23 ± 22	-
-Mean heart rate, bpm	66 ± 11	65 ± 12	67 ± 9	-
**Symptoms:**				
-Snoring	66 (57)	42 (57)	24 (57)	0.826
-Witnessed Apneas	9 (8)	3 (4)	6 (14)	0.065
-Unrefreshing Sleep	20 (17)	11 (15)	9 (21)	0.433
-Fatigue	32 (28)	17 (23)	15 (36)	0.118
-Sleepiness behind the wheel	8 (7)	6 (8)	2 (5)	0.711
**Questionnaires:**				
-ESS Score	5.7 ± 3.8	5.1 ± 3.7	6.7 ± 3.9	0.047
-ESS > 10	11 (9)	5 (7)	6 (14)	0.203
-Positive Berlin Questionnaire	48 (41)	27 (36)	21 (50)	0.098

**Table 4:** Multivariate logistic regression results for OSA (AHI ≥ 15). Bold indicates *p*-value < 0.05.

Variable	Odds Ratio	95% CI	*p*-Value
Age/10	1.64	1.03–2.61	0.036
Witnessed Apneas	6.20	1.31–29.22	0.021
Sex	0.37	0.13–1.07	0.066
Body Mass Index	1.09	0.98–1.21	0.098
Hospitalization Length	1.06	1.00–1.14	0.061

## Data Availability

The clinical dataset generated and analyzed during the current study is securely archived in the institutional electronic database in compliance with applicable privacy and data protection regulations. The anonymized minimal dataset supporting the findings of this study is provided as Supplementary Material (Dataset S3). It includes only de-identified variables required for replication of the main analyses. No information that could allow patient re-identification is included.

## Supplementary Materials

The following supporting information can be downloaded at: https://www.mdpi.com/article/10.3390/jcm14238332/s1, Table S1. Comparison of model performance using the conventional 0.50 cut-off and the optimal Youden-derived threshold. Metrics include sensitivity, specificity, accuracy, positive predictive value (PPV), negative predictive value (NPV). Table S2. Performances of the regressive model with alternative diagnostic thresholds for OSA, defined as AHI ≥ 5, ≥15, and ≥30 events·h^−1^. Figure S1. Comparison of ROC curves for the predictive model across different AHI thresholds. ROC curves showing the discriminative performance of the logistic regression model in detecting OSA defined using three alternative AHI thresholds (≥5, ≥15, and ≥30 events·h^−1^). Dataset S3. Anonymized minimal dataset.

## Abbreviations

The following abbreviations are used in this manuscript:

OSA	Obstructive sleep apnea
CPAP	Continuous positive airway pressure
PSG	Polysomnography
HSAT	Home sleep apnea testing
BQ	Berlin Questionnaire
ESS	Epworth Sleepiness Scale
TIA	Transient ischemic attack
BMI	Body mass index
NIHSS	National Institutes of Health Stroke Scale
mRS	Modified Rankin Scale
PFO	Patent foramen ovale
AF	Atrial fibrillation
ESUS	Embolic stroke of undetermined source
AASM	American Academy of Sleep Medicine
AHI	Apnea–hypopnea index
ODI	Oxygen desaturation index
T90%	Percentage of total recording time spent with SpO_2_ < 90%
SD	Standard deviation
OR	Odds ratio
CI	Confidence interval
ROC	Receiver operating characteristic
AUC	Areas under the curve
