# Supplementary material for: Age and Witnessed Apneas as Independent Predictors of Obstructive Sleep Apnea After Stroke: A Prospective Cohort Study
Source: J Clin Med. 2025 Nov 24;14(23):8332. doi: 10.3390/jcm14238332 (PMC12693645; doi:10.3390/jcm14238332)
Supplement: Supplementary file 1 [file jcm-14-08332-s001.zip › Figure S1 - supplementary.pdf]

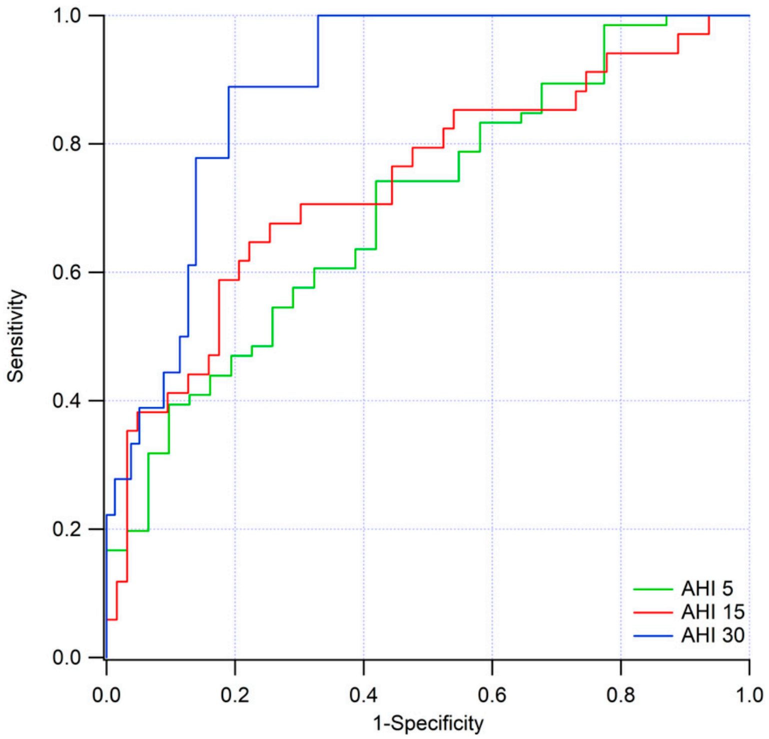

Figure S1. Comparison of ROC curves for the predictive model across different AHI thresholds.
ROC curves showing the discriminative performance of the logistic regression model in detecting OSA defined using three alternative AHI thresholds (≥5, ≥15, and ≥30 events·h^{-1}).
