# Supplementary material for: Age and Witnessed Apneas as Independent Predictors of Obstructive Sleep Apnea After Stroke: A Prospective Cohort Study
Source: J Clin Med. 2025 Nov 24;14(23):8332. doi: 10.3390/jcm14238332 (PMC12693645; doi:10.3390/jcm14238332)
Supplement: Supplementary file 1 [file jcm-14-08332-s001.zip › Table S1 - supplementary.pdf]

Model	Cut-off	Sensitivity	Specificity	Accuracy	PPV	NPV	AUC (95% CI)
Regression model	0.50	41.2%	90.3%	72.9%	70.0%	73.7%	0.739 (0.630–0.847)
	0.385	64.7%	77.8%	73.2%	61.1%	80.3%	
Berlin	0.50	11.4%	93.8%	64.6%	50.0%	65.9%	0.596 (0.478–0.715)
	0.354	60.0%	57.8%	58.6%	43.8%	72.5%	
ESS	0.50	12.5%	94.3%	64.5%	55.6%	65.3%	0.616 (0.498–0.733)
	0.404	42.5%	80.0%	66.4%	54.8%	70.9%	

Table S1. Comparison of model performance using the conventional 0.50 cut-off and the optimal Youden-derived threshold. Metrics include sensitivity, specificity, accuracy, positive predictive value (PPV), negative predictive value (NPV).
