# Supplementary material for: Age and Witnessed Apneas as Independent Predictors of Obstructive Sleep Apnea After Stroke: A Prospective Cohort Study
Source: J Clin Med. 2025 Nov 24;14(23):8332. doi: 10.3390/jcm14238332 (PMC12693645; doi:10.3390/jcm14238332)
Supplement: Supplementary file 1 [file jcm-14-08332-s001.zip › Table S2 - supplementary.pdf]

Threshold	Sensitivity	Specificity	Accuracy	PPV	NPV	AUC (95% CI)
**AHI ≥ 5**	89.4%	22.6%	68.0%	71.1%	50.0%	0.702 (0.593–0.810)
**AHI ≥ 15**	41.2%	90.3%	72.9%	70.0%	73.7%	0.738 (0.630–0.846)
**AHI ≥ 30**	38.9%	93.7%	83.5%	58.3%	87.1%	0.888 (0.822–0.955)

Table S2. Performances of the regressive model with alternative diagnostic thresholds for OSA, defined as AHI ≥5, ≥15, and ≥30 events·h^{-1}.
